# Radiation-Free Percutaneous Coronary Intervention: Myth or Reality?

**DOI:** 10.3390/jcdd12090339

**Published:** 2025-09-03

**Authors:** Sotirios C. Kotoulas, Andreas S. Triantafyllis, Nestoras Kontogiannis, Pavlos Tsinivizov, Konstantinos Antoniades, Ibraheem Aqeel, Eleni Karapedi, Angeliki Kolyda, Leonidas E. Poulimenos

**Affiliations:** Department of Interventional Cardiology, Asklepeion General Hospital, 16673 Athens, Greece; soter96@icloud.com (S.C.K.); kontonest@gmail.com (N.K.); pavlost1980@gmail.com (P.T.); konstantinos.a@hotmail.com (K.A.); ibraheemaqeel@yahoo.com (I.A.); karapedi.eleni@gmail.com (E.K.); kolyange@yahoo.gr (A.K.); leonp@otenet.gr (L.E.P.)

**Keywords:** radiation-free PCI, robotic PCI, protective equipment, catheterization laboratory, novel techniques, coronary artery disease

## Abstract

**Background:** Radiation exposure in the cardiac catheterization laboratory remains a critical occupational hazard for interventional cardiologists and staff, contributing to orthopedic injuries, cataracts, and malignancy. In parallel, procedural complexity continues to increase, demanding both precision and safety. Robotic-assisted percutaneous coronary intervention (R-PCI), alongside advanced shielding systems and imaging integration, has emerged as a transformative strategy to minimize radiation and enhance operator ergonomics. **Objective:** This state-of-the-art review synthesizes the current clinical evidence and technological advances that support a radiation-reduction paradigm in percutaneous coronary intervention (PCI), with a particular focus on the role of R-PCI platforms, procedural modifications, and emerging shielding technologies. **Methods:** We reviewed published clinical trials, registries, and experimental studies evaluating robotic PCI platforms, contrast and radiation dose metrics, ergonomic implications, procedural efficiency, and radiation shielding systems. Emphasis was given to the integration of CT-based imaging (coronary computed tomography angiography—CCTA, fractional flow reserve computed tomography—FFR-CT) and low-dose acquisition protocols. **Results:** R-PCI demonstrated technical success rates of 81–100% and clinical success rates up to 100% in both standard and complex lesions, with significant reductions in operator radiation exposure (up to 95%) and procedural ergonomic burden. Advanced shielding technologies offer radiation dose reductions ranging from 86% to nearly 100%, while integration of (CCTA), (FFR-CT), and Artificial Intelligence (AI) -assisted procedural mapping facilitates further fluoroscopy minimization. Robotic workflows, however, remain limited by lack of device compatibility, absence of haptic feedback, and incomplete integration of physiology and imaging tools. **Conclusions:** R-PCI, in combination with shielding technologies and imaging integration, marks a shift towards safer, radiation-minimizing interventional strategies. This transition reflects not only a technical evolution but a philosophical redefinition of safety, precision, and sustainability in modern interventional cardiology.

## 1. Introduction

Cardiovascular disease remains the leading cause of mortality in Europe, responsible for approximately 3.8 million deaths annually, with ischemic heart disease accounting for nearly half of these fatalities [1]. Since Andreas Grüntzig’s first percutaneous coronary intervention (PCI) in 1977, the field has witnessed a continuous evolution towards less invasive, more effective revascularization strategies [2,3]. PCI has become the treatment of choice for obstructive coronary artery disease, offering rapid symptom relief and improved outcomes in both elective and acute settings. Despite major advances in pharmacotherapy, stent design, and procedural imaging, the delivery of PCI still imposes substantial health risks on operators and staff within cardiac catheterization laboratories. Prolonged exposure to ionizing radiation has been linked to a range of deterministic and stochastic effects, including cataracts, hematologic alterations, and a higher risk of left-sided brain tumors [4,5,6]. Moreover, chronic use of heavy lead-based personal protective equipment (PPE) contributes to orthopedic injuries, reported in up to 20% of cath-lab personnel [7,8]. These occupational hazards persist even with current shielding protocols, leading to increasing calls for structural innovation in procedural delivery.

Introduced in 2005, robotic-assisted PCI (R-PCI) emerged as a potential solution to mitigate radiation exposure and musculoskeletal strain by enabling operators to perform interventions from radiation-shielded control stations [9,10,11]. While robotic assistance is well-established in surgical specialities, its uptake in interventional cardiology has been more measured. Early skepticism stemmed from concerns about device compatibility, procedural efficiency, and limited tactile feedback. However, successive generations of robotic platforms have demonstrated improved precision, reduced operator radiation dose, and expanding clinical feasibility in a variety of lesion subsets [12,13,14,15]. Importantly, R-PCI is no longer viewed solely as a tool for operator protection. It is increasingly recognized as a key component in the broader paradigm shift towards precision-guided, low-radiation interventional cardiology. Advances in robotics, imaging integration, artificial intelligence, and remote connectivity are converging toward a new standard of care that emphasizes patient-specific planning, procedural automation, and minimized occupational risk [16,17,18].

**Figure 1:**
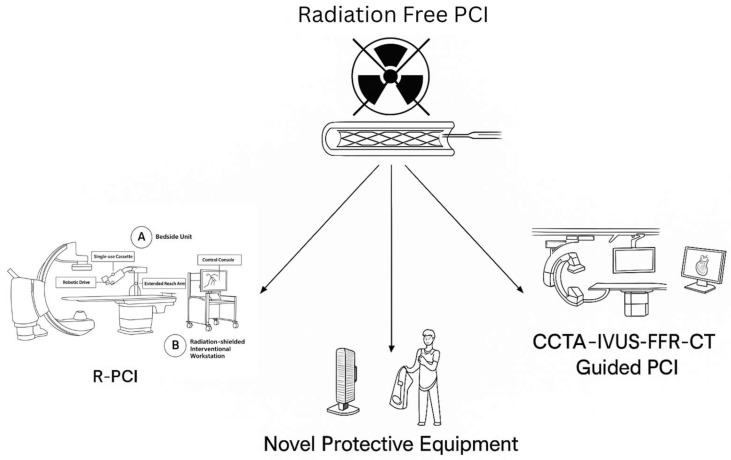
Aspects of radiation-free PCI.

In this narrative review, we examine the concept, evolution, and clinical performance of R-PCI as well as novel radiation shielding technologies with the aim of achieving radiation-free PCI in the future, as shown in Figure 1. Studies of varying design and sample size were included to provide a broad overview of the field, without direct quantitative comparison between heterogeneous datasets. We evaluate technical features, safety outcomes, procedural limitations, and integration with adjunctive technologies, and we explore its future potential within a progressively digital and ergonomic catheterization lab environment.

### Methods

This is a narrative review synthesizing peer-reviewed literature on robotic and radiation-reduction strategies in percutaneous coronary intervention (PCI). We conducted a structured literature search in PubMed/MEDLINE, Scopus, and Web of Science for studies published from January 2010 to June 2025 using keywords including “robotic PCI,” “radiation-free PCI,” “coronary computed tomography angiography,” and “fractional flow reserve computed tomography”. Reference lists of key articles were also reviewed. Inclusion criteria were English-language studies reporting clinical outcomes, procedural metrics, safety data, or cost-effectiveness related to robotic PCI or radiation-minimization strategies. Exclusion criteria were non-human studies, conference abstracts without full-text articles, and studies not relevant to interventional cardiology. As this is a narrative rather than a systematic review, we did not perform quantitative pooling or meta-analysis.

## 2. Discussion

### 2.1. Robotic PCI

**Figure 2:**
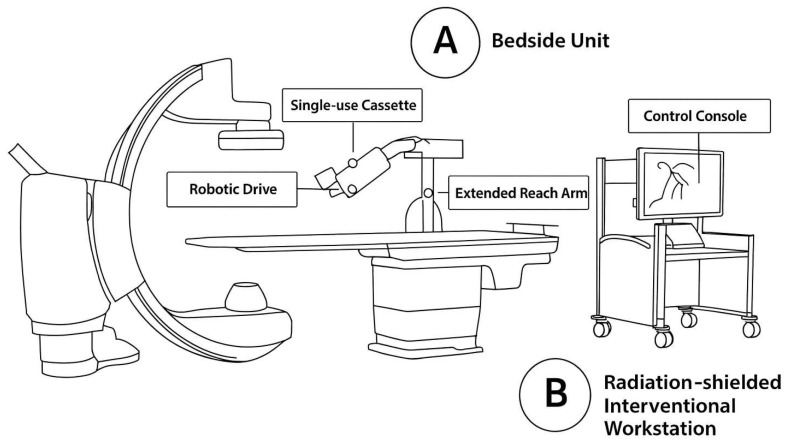
Robotic-PCI system.

A robotic percutaneous coronary intervention (R-PCI) system employs a specialized system consisting of a radiation-shielded cockpit for remote operation, an articulated robotic arm, and a sterile disposable cassette (Figure 2). The procedure begins with the operator manually obtaining arterial access and positioning the guide catheter. Subsequently, control is transferred to the robotic system. From within the cockpit—equipped with high-resolution monitors and precision joysticks—the operator remotely advances guidewires, inflates balloons, and deploys stents under angiographic guidance. The robotic arm manipulates the interventional devices through the sterile cassette, which serves as the interface for the advancement and retraction of equipment [13].

**Figure 3:**
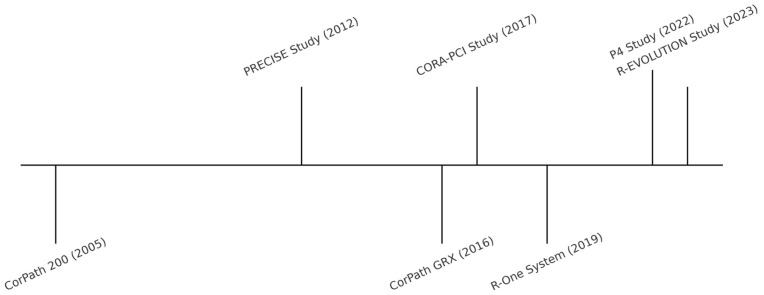
Timeline of R-PCI platforms and key studies.

R-PCI technology has advanced significantly since its inception. Early platforms, such as the Corindus CorPath 200 (Corindus, Waltham, MA, USA), introduced a robotic arm controlled from a shielded cockpit, enabling operators to manipulate guidewires and devices remotely, reducing radiation exposure and improving ergonomic safety [9,12]. Subsequent advancements, including the Corindus CorPath GRX (Corindus, Waltham, MA, USA), added guiding catheter control, automated wiring techniques like spin, wiggle, and rotate-on-retract, and improved precision through artificial intelligence-enhanced movement patterns [14]. These innovations allow for greater accuracy, enhanced procedural efficiency, and the ability to tackle increasingly complex coronary lesions. The R-One (Robocath, Rouen, France) platform further simplified the system setup, reducing the learning curve. Figure 3 outlines the chronological evolution of robotic PCI platforms alongside key clinical trials [15]. Despite these advancements, limitations remain, such as incompatibility with rotational atherectomy devices and over-the-wire systems, double guidewire techniques (e.g., bifurcation PCI), and limitations in tactile feedback—highlighting areas for further technological development [19]. Nevertheless, recent series have demonstrated the feasibility of R-PCI in bifurcation lesions, with high technical success when supported by careful case selection and adjunctive techniques [20]. These iterative enhancements continue to expand the scope and safety of robotic PCI procedures.

#### 2.1.1. Safety and Feasibility

Robotic PCI (R-PCI) has demonstrated high technical and clinical success rates, particularly for complex coronary lesions. Studies such as CORA-PCI and PRECISE revealed success rates ranging from 93.3% to 100% for clinical success and 81% to 98.8% for technical success, with clinical success consistently defined as successful lesion treatment without major adverse cardiovascular events (MACEs) [10,11]. Compared to manual PCI (M-PCI), this equivalency in outcomes highlights its safety and efficacy. Data from four trials involving 357 patients treated with the first-generation Corindus CorPath 200 platform reported technical success rates between 91.7% and 98.8% and clinical success rates ranging from 97.6% to 100% [12,13]. Transitioning to the second-generation Corindus CorPath GRX platform, Smitson et al. evaluated 40 patients—77.8% of whom had type B2/C lesions—and reported a 90% technical success rate. Clinical success was achieved in 97.5% of cases [14]. Notable procedural challenges included one chronic total occlusion (CTO) that could not be crossed either robotically or manually, a case requiring a buddy wire to advance a stent through previously placed struts, and a long, 95% stenosed segment where the robotic system could not achieve wire crossing—likely due to suboptimal grip in the robotic cassette. Another balloon-uncrossable lesion required escalation to orbital atherectomy [21].

Hirai et al. [15] retrospectively assessed the Corindus CorPath GRX platform in a single center, in patients undergoing PCI for a single CTO lesion using a hybrid approach. Following manual lesion crossing with microcatheters and CTO-specific tools, the guidewire was exchanged for a workhorse wire, and the robotic system was used for completion [15]. Ninety-eight percent of the planned robotic cases were completed successfully according to the prespecified hybrid protocol. One case required unplanned manual intervention due to thrombus formation, treated with intracoronary tissue plasminogen activator (tPA). Lesion complexity was comparable between robotic and manual groups (J-CTO Score: 2.1 ± 1.1 vs. 2.0 ± 1.2; *p* = 0.81), with no significant difference in MACE rates [15]. Zyśk et al. highlighted the potential of CT-derived scoring systems to predict procedural success in CTO PCI using hybrid algorithms, underscoring the importance of imaging-based planning in these complex subsets [21]. Further data from five additional single-center studies (*n* = 313) using the Corindus CorPath GRX platform revealed technical success ranging from 81% to 98% and clinical success between 93.3% and 100% [14,15,22,23,24].

The European multicenter R-EVOLUTION prospective registry, which investigated the Robocath R-One system, reported 95.2% technical and 100% clinical success in 62 patients with de novo coronary artery stenosis. A quarter of lesions were type B2/C, and the procedure was performed using radial access in 96.8% of cases [16]. Across all these studies, conversions from R-PCI to M-PCI occurred infrequently and were largely driven by procedural limitations rather than complications. Common reasons included inadequate guide catheter support, failure to deliver balloons or stents, and the need for advanced tools such as guide extension catheters, buddy wires, or atherectomy devices [10,11,15,25]. Technical challenges specific to the robotic platform—particularly limitations in wire manipulation, the absence of tactile feedback, and resistance encountered during device delivery—were the predominant causes for procedural crossover or partial manual assistance in contemporary studies [10,11,15,25]. Adverse-event-driven conversions were rare, and conversion times, when reported, were typically under one minute [15]. Table 1 provides a concise comparative overview of major clinical trials and registries in robotic PCI, including study design, outcomes, and key limitations.

#### 2.1.2. Radiation Exposure Reduction

R-PCI, as shown in the R-EVOLUTION trial by Durand et al., ensures radiation protection. Compared to manual PCI, the robotic intervention led to a 69% reduction in operator exposure and a 31% reduction in patient dose, thanks to the remote-controlled intervention and improved shielding [16]. The use of a robotic cockpit eliminates the need for personal lead aprons and improves procedural ergonomics, especially in high-volume centers [9,26]. In addition, the PRECISE study, a prospective, multicenter trial, demonstrated a 95% reduction in radiation exposure to the primary operator with the use of the Corindus CorPath GRX R-PCI system [11].

### 2.2. Imaging Modalities’ Integration

Integrating advanced imaging modalities such as coronary computed tomography angiography (CCTA) and fractional flow reserve computed tomography (FFR-CT) represents a transformative approach to percutaneous coronary intervention (PCI). These technologies enhance pre-procedural planning and real-time guidance, offering precise anatomical and functional assessments that can improve procedural strategy. CCTA provides high-resolution 3D reconstructions of the coronary tree, allowing operators to evaluate plaque morphology, lesion length, and vessel dimensions with submillimeter accuracy. FFR-CT complements this anatomical view with a non-invasive assessment of lesion-specific ischemia, enabling targeted interventions that address physiologically significant stenoses [27,28]. Beyond FFR-CT, CCTA-derived scoring models have also been shown to predict chronic total occlusion (CTO) PCI outcomes with high accuracy, supporting their role in procedural planning and patient selection [20].

When integrated with real-time angiography, these modalities provide a dynamic roadmap for lesion navigation, potentially reducing the need for repeated angiographic imaging and associated radiation exposure. Low-dose acquisition protocols, low frame-rate fluoroscopy, and AI-enhanced image processing can further decrease exposure for both patient and operator. The ongoing P4 study, a randomized, multicenter trial, is directly evaluating whether CT-guided PCI can match or surpass intravascular ultrasound (IVUS)-guided PCI in procedural and clinical outcomes, with post-PCI FFR as a primary endpoint [27].

However, in the current era, CCTA/FFR-CT integration is not yet a full substitute for invasive intravascular imaging. IVUS and optical coherence tomography (OCT) remain the gold standards for intraprocedural assessment of stent expansion, apposition, and plaque modification, offering unmatched spatial resolution and tissue characterization in real time [29,30]. While CCTA can guide lesion selection and stent sizing, its role in confirming optimal stent deployment remains limited compared with IVUS and OCT, which can immediately guide corrective measures.

Several barriers limit routine CCTA/FFR-CT adoption for PCI planning. These include spatial co-registration errors between CT-derived models and live fluoroscopy (affected by patient movement, cardiac phase variation, or anatomical complexity), variability in image quality due to arrhythmia, high heart rates, or heavy calcification, and inconsistent reimbursement frameworks across healthcare systems [27,28]. Additionally, incorporating CT-based planning into the workflow adds processing time and may require dedicated software and trained personnel. The current evidence base is largely composed of single-center experiences and observational registries, which limits the generalizability of findings. Large-scale, multicenter randomized controlled trials are still required to establish the long-term clinical impact, cost-effectiveness, and sustainability of these technologies. Until such data are available, CCTA/FFR-CT should be viewed as a promising adjunct to—rather than a replacement for—invasive imaging, with integration tailored to patient selection, institutional resources, and operator expertise.

### 2.3. Robotic PCI and Imaging Integration

Synchronizing CT data with the fluoroscopic C-arm not only limits radiation exposure but also reduces the use of contrast media, making procedures safer and more efficient [28,30]. Early registry data further suggest that combining R-PCI with CCTA-guided planning may not only improve clinical outcomes but also favorably impact hospital economics [31].The combined use of these technologies profoundly impacts outcomes, especially in complex or high-risk cases. FFR-CT allows operators to simulate post-PCI physiological outcomes before the procedure, aiding in selecting strategies and materials that maximize therapeutic benefits. This is particularly valuable in diffuse coronary artery disease, where focal interventions may not adequately restore blood flow, or in borderline lesions, where physiological guidance confirms the necessity of intervention [32].

### 2.4. Protective Equipment and Emerging Radiation Shielding Technologies

Advances in protective equipment for the cardiac catheterization laboratory have significantly improved radiation safety while addressing long-standing ergonomic challenges for operators. Modern solutions focus on maintaining or improving shielding efficacy while reducing physical strain. Lightweight aprons made from composite materials such as tungsten, bismuth, and titanium provide comparable radiation attenuation at 20–40% less weight than conventional lead, easing the physical demands of long procedures. Redesigned thyroid collars and leaded eyewear enhance fit and lateral coverage, reducing the risk of cataracts and thyroid malignancy without compromising comfort [4,7].

Room-based shielding systems, including ceiling-suspended upper body shields and table-mounted lower-body curtains, can block up to 90% of scatter radiation and reduce reliance on personal lead. More advanced systems, such as the Zero-Gravity suspended shield, completely offload the apron’s weight from the operator, improving posture and reducing fatigue during prolonged interventions [33].

Recent apron-free, operator-enclosure systems have set new standards for cath-lab radiation protection. The Rampart IC (Rampart, Alabama, USA) system, a mobile floor-mounted lead–acrylic barrier, encloses the operator and has demonstrated up to a 99.7% reduction in radiation exposure in randomized trials [34]. The Protego system (Idi diagnostics, Fitchburg, MA, USA) combines rigid and flexible shielding to protect both operator and assistant, with reductions of 95.9–99.8% at key anatomical sites [32]. The StemRad MD (Stemrad, Tampa, FL, USA) exoskeleton integrates full-body shielding with musculoskeletal support, redistributing weight through the hips and torso and reducing cranial, ocular, and thyroid dose by >90% in benchtop/clinical dosimetry reports [26,35].

These systems, when combined with optimized procedural techniques and radiation-sparing workflows, represent a paradigm shift toward eliminating the orthopedic toll of traditional Personal Protective Equipment (PPE) while substantially reducing radiation burden. Their implementation, particularly in high-volume centers, supports not only operator health but also procedural efficiency, contributing to a safer and more sustainable working environment in interventional cardiology.

### 2.5. Radiation Exposure Reduction in Clinical Trials

While the pursuit of radiation-free PCI remains a long-term goal, significant strides have already been made in reducing radiation exposure through technological and procedural innovation. Multiple studies have demonstrated impressive radiation reduction without compromising procedural outcomes and safety. The REDUCE-PCI study implemented real-time dosimetry, staff education, and shielding protocols, resulting in a 45% reduction in operator radiation dose during PCI procedures [36]. Complementary technologies such as the Zero-Gravity system and ceiling-mounted protective shields have shown a 90–95% decrease in operator head and upper body exposure, offering both radiation protection and ergonomic support [33]. Gupta et al. tested a low-frame-rate imaging protocol (3.8 + 7.5 fps) and reduced cine runs in elective stenting, achieving an 80% reduction in total radiation dose with no significant compromise in image quality or procedural success [37]. Similarly, the PROTECTION VIII multicenter observational registry showed that integration of flat-panel detectors, optimized collimation, and low-dose settings led to a 47% reduction in the median dose-area product (DAP) across European centers [38]. Further insights come from trials focused on robotic PCI. Patel et al. [22] in a large propensity-score-matched cohort of 560 patients (280 R-PCI vs. 280 M-PCI), reported significantly lower radiation exposure in the R-PCI group. Mean air kerma was reduced from 1110 (M-PCI) to 884 mGy (R-PCI), and dose area product from 5746 to 4734 cGy·cm^2^, respectively (*p* < 0.01 for both) [22]. Hirai et al. found similarly favorable results in a CTO-PCI cohort, with significantly shorter fluoroscopy times (37.9 vs. 48.6 min; *p* < 0.01) and reduced air kerma (1522 vs. 2466 mGy; *p* < 0.01) in R-PCI compared to M-PCI [15]. Although early studies such as those by Beyar et al. and Smilowitz et al. found no statistically significant differences in fluoroscopy time or radiation dose [9,39], subsequent studies suggest that operator training and platform evolution significantly influence exposure metrics. Patel et al. notably demonstrated that patients treated later in their R-PCI experience received substantially less radiation, underscoring the role of experience and protocol refinement. Dose area product declined from 967.3 ± 863.2 to 361 ± 231.1 (*p* = 0.01) as procedural familiarity improved [23]. Robotic platforms, coupled with imaging integration and operator experience, are key enablers in the evolution toward radiation-free interventional cardiology. As summarized in Table 2, various clinical trials and device evaluations have reported significant reductions in operator and patient radiation exposure.

### 2.6. Contrast Use and Procedural Efficiency

Several studies have evaluated contrast volume and procedural time in robotic versus manual PCI, offering insight into the operational efficiency of emerging robotic platforms. Overall, findings suggest comparable contrast use across techniques, with procedural times showing a mild increase in robotic cases—particularly during the learning curve phase. Smilowitz et al. [9] found no significant difference in contrast volume between patients undergoing R-PCI and M-PCI (121 ± 47 mL vs. 137 ± 62 mL, respectively; *p* = 0.11). Similarly, Madder et al. reported near-equivalent volumes (167 ± 89 mL for R-PCI vs. 145 ± 92 mL for M-PCI; *p* = 0.12) [12]. In the CORA-PCI study, although initial findings suggested a trend toward lower contrast use with robotic intervention (183.4 ± 78.7 mL vs. 202.5 ± 74 mL), this difference was not statistically significant in the propensity-matched analysis [11]. In a CTO-specific analysis, Hirai et al. reported similar contrast volumes (111 ± 39 mL in R-PCI vs. 118 ± 53 mL in M-PCI; *p* = 0.47) [15]. Patel et al., in a large 1:1 propensity-matched cohort of 560 patients, confirmed no significant difference in contrast use between the two groups (140 [100–180] mL for R-PCI vs. 130 [103–170] mL for M-PCI; *p* = 0.905) [22].

With respect to procedural duration, early studies reported slightly longer times for R-PCI. Beyar et al., in a small cohort of patients undergoing single-lesion PCI, found no significant difference (R-PCI: 44 ± 32.7 min vs. M-PCI: 61 ± 19 min; *p* = ns) [39]. In contrast, Madder et al. reported significantly prolonged durations in R-PCI cases (55 [22.0] min) compared to M-PCI (45 [37.0] min; *p* = 0.02) [12]. In the CORA-PCI study, R-PCI procedures lasted longer even after matching (43 ± 26 min vs. 34 ± 17 min; *p* = 0.007) [11]. Patel et al. also observed significantly increased procedural times with robotic systems (37 [min vs. 27 min; *p* < 0.0005) [22]. Interestingly, Hirai et al. found similar total procedural durations in their CTO cohort (89.6 ± 27.1 min for R-PCI vs. 93.4 ± 30.5 min for M-PCI; *p* = 0.52) [15]. However, the robotic phase of the intervention—from guidewire exchange to guide catheter removal—was significantly longer in R-PCI (40.6 ± 12.7 min vs. 32.1 ± 17.8 min; *p* < 0.01), accounting for a greater proportion of total procedural time (47.8% vs. 35.5%; *p* < 0.03). Notably, procedural efficiency with robotic systems appears to improve with experience. In the R-EVOLUTION study, centers with prior experience of more than five R-PCI procedures demonstrated significantly shorter overall procedure times (35.5 ± 11.1 min) than less experienced centers (45.3 ± 16.6 min; *p* = 0.01) [16]. This reinforces the importance of operator familiarity and system training in optimizing robotic workflow and time efficiency. Table 3 and Table 4 present comparative data on contrast use, procedural times, and clinical/technical success rates between robotic and manual PCI.

### 2.7. Limitations

Despite the demonstrated feasibility and safety of robotic-assisted PCI (R-PCI), several limitations continue to hinder its broader clinical adoption. One of the primary challenges is the limited ability of current robotic platforms to escalate treatment in complex interventions. In multiple studies, procedural crossover from R-PCI to manual PCI was necessitated by lesion resistance, failure to deliver devices, or the need for advanced support tools such as buddy wires or guide extension catheters. Notably, the absence of tactile feedback—an essential sensory input for operators—can impair navigation through tortuous or calcified anatomy. In the PRECISE trial, inability to cross a long, severely stenosed lesion was attributed to suboptimal wire grip within the robotic cassette [10]. Similarly, conversions due to insufficient guide catheter support were reported in CORA-PCI and R-EVOLUTION, though second-generation systems such as CorPath GRX offer partial mitigation via remote guide catheter manipulation [11,16]. Despite these iterative improvements, industry support has not been uniform. In 2023, Siemens Healthineers, discontinued production of its Corindus-based robotic PCI platform for coronary applications and has redirected the technology primarily toward neurointerventional procedures, according to an industry announcement [40].

Another critical limitation is the lack of compatibility with intracoronary imaging and physiology tools. While studies have shown the manual feasibility of using IVUS and OCT during R-PCI [11,18,30,36,39], there is currently no regulatory-approved robotic control of these devices, nor of pressure wires for invasive FFR assessment—tools that are essential in contemporary PCI, particularly for complex or ambiguous lesions [29,30]. Lesion crossing in chronic total occlusion (CTO) cases remains another barrier. In the study by Hirai et al., all CTO lesions were crossed manually before switching to robotic control, reflecting the incompatibility of the CorPath GRX platform with over-the-wire systems, microcatheters, and atherectomy devices [15]. Moreover, even in successfully crossed lesions, balloon or stent delivery sometimes failed under robotic control, requiring manual conversion or the use of adjunctive techniques [14].

Operator training and team experience are additional limiting factors. The learning curve associated with R-PCI is non-trivial. In the R-EVOLUTION trial, procedures were significantly longer in centers with less experience [16], while Patel et al. observed a notable reduction in fluoroscopy time between early and later robotic cases, highlighting the impact of procedural familiarity [23]. Finally, robotic PCI platforms introduce workflow inefficiencies that can affect their implementation in routine practice. Setup time for robotic arms and cassettes, limited device compatibility, and frequent need for manual intervention during certain procedural steps can disrupt cath lab dynamics—particularly in low-volume centers or in urgent scenarios. Tasks such as catheter exchanges, post-dilation with specialty balloons, or bifurcation wiring often fall outside the robotic workflow and require operator re-engagement at the table [22]. Until robotic systems achieve broader hardware compatibility and greater integration into standard interventional workflows, their use may remain restricted to high-volume centers with the necessary infrastructure and trained personnel.

Further limitations are related to the economic and logistical barriers that may impede widespread adoption of R-PCI. The acquisition cost of a robotic platform, often exceeding USD 500,000–600,000, is compounded by ongoing expenses for disposable cassettes and annual maintenance contracts, which can be prohibitive for low- and moderate-volume centers [41]. While preliminary cost-effectiveness models suggest potential long-term savings through reduced occupational injury, lower radiation-related health risks, and extended operator career longevity [41], these benefits remain largely theoretical, with no large-scale, real-world economic evaluations currently available. Furthermore, reimbursement frameworks in most health systems do not offset the capital and consumable costs of robotic PCI, limiting its financial viability [41]. Practical considerations—including the need for dedicated installation space, integration with existing cath lab infrastructure, and specialized operator/staff training—pose additional barriers. The learning curve, as documented in multiple registries [17,23], can result in longer procedural times and reduced throughput during early implementation phases, creating challenges for centers under procedural efficiency pressures. These real-world constraints suggest that early adoption is most feasible in high-volume tertiary centers with adequate infrastructure, experienced teams, and institutional commitment to long-term technology integration.

### 2.8. The Philosophical Shift in Interventional Cardiology

Over the past decade, interventional cardiology has witnessed a progressive transformation—shifting from a fluoroscopy-dominated, manually intensive discipline to one increasingly shaped by precision, safety, and technological integration. This evolving philosophy is no longer centered solely on technical success or angiographic endpoints but emphasizes operator safety, ergonomic sustainability, radiation minimization, and patient-centered innovation. At the core of this transformation lies the concept of radiation awareness as a fundamental operator responsibility. The longstanding reliance on personal lead aprons and conventional shielding is being re-evaluated, considering mounting evidence linking chronic radiation exposure to orthopedic injury, malignancy, and cataracts in cath lab personnel [4,5,7,8]. This awareness has catalyzed the adoption of robotic PCI systems, advanced shielding technologies, and procedural innovations that reduce reliance on continuous fluoroscopic imaging.

The introduction of robotic PCI epitomizes this shift. These systems offer not only mechanical precision and control but also a philosophical reorientation, placing the operator at a safe distance from the radiation source while preserving procedural effectiveness. Operators no longer stand in lead aprons under the gantry but instead sit in shielded cockpits or behind remote-control consoles, embracing a model of care that values long-term occupational health. Beyond robotics, the integration of pre-procedural imaging (CCTA, FFR-CT) and the use of AI-assisted procedural planning tools represent a shift toward data-enriched, image-guided PCI. This reduces intra-procedural radiation while allowing real-time decisions to be based on physiology, anatomy, and computational modeling—replacing guesswork with precision [28,30,41]. Along with these technological advances, a systemic shift in culture has emerged. Institutions now prioritize radiation-reduction protocols, operator retraining, and ergonomic design of catheterization labs. Quality improvement programs—such as those assessed in the study by Roguin et al.—demonstrate that multidisciplinary engagement can reduce both patient and operator radiation doses by more than 50% without sacrificing outcomes [36]. This evolving mindset has also influenced how procedural success is defined. Rather than focusing purely on angiographic success, modern interventionalists consider patient safety, minimal contrast use, reduced procedure duration, and long-term outcomes, including operator wellness. In this model, innovation is not just technical—it is ethical and humanistic, aligning with a broader healthcare movement toward sustainable, evidence-driven practice. It is a paradigm shift—a reflection of changing values in interventional cardiology. It challenges operators to think beyond the angiogram, to prioritize safety without compromise, and to embrace innovation not merely for its novelty, but for its ability to reshape the field for both patient and provider.

### 2.9. Future Aspects

The evolution of robotic-assisted PCI (R-PCI) represents a significant milestone in the ongoing transformation of interventional cardiology toward precision, safety, and sustainability. However, for R-PCI to reach its full potential and achieve widespread clinical adoption—particularly as part of radiation-free procedural strategies—several technical, workflow, and structural limitations must be addressed. The future development of R-PCI platforms should focus on enhancing device compatibility, integrating physiological and imaging tools, improving user interaction, and enabling remote capabilities. One of the most pressing needs is the expansion of device compatibility. Current robotic systems remain limited in their ability to interface with over-the-wire systems, dual-lumen catheters, microcatheters, and atherectomy devices—all essential tools for complex interventions and chronic total occlusions (CTOs) [11,42]. Additionally, robotic incompatibility with intravascular imaging (IVUS, OCT) and physiology assessment tools (FFR/iFR) precludes full procedural automation. Although manual use of such modalities has been demonstrated in R-PCI settings [18,29], robotic platforms must evolve to permit seamless, operator-controlled deployment of these technologies to fulfill guideline-recommended practice and enhance outcomes in complex PCI [25,29]. Furthermore, the absence of tactile feedback remains a critical limitation in the current generation of robotic platforms. Experienced operators rely heavily on haptic cues during lesion crossing, particularly in tortuous or calcified anatomy. The development of force-sensing actuators and real-time haptic feedback systems could substantially enhance procedural safety and operator confidence. Early applications of such systems in neurointerventional and endovascular robotics show promise and should be adapted for coronary use [43,44].

Recent work demonstrates AI-assisted lesion assessment and guidewire navigation, improving planning and procedural precision, with potential to automate complex steps in R-PCI systems [45]. AI enabled navigation also holds great potential. Recent studies have demonstrated AI-assisted lesion assessment, guidewire navigation, and prediction of wire passage trajectories based on imaging inputs [45]. Incorporating such technologies into robotic platforms could improve procedural planning, automate complex navigation steps, and reduce fluoroscopy time further. The combination of AI and robotics is particularly relevant in diffuse coronary artery disease, where treatment planning based on CT-derived physiology and plaque characteristics can help tailor interventions more precisely. User interface design and workflow optimization are also essential areas for future advancement. Robotic PCI platforms must evolve toward more intuitive control systems with modular designs, simplified cassette preparation, and integration of key tools (imaging, contrast injection, physiology) into a single cockpit interface. Emerging concepts such as voice-activated commands, gesture control, and real-time intraprocedural 3D imaging overlays could further enhance ergonomic efficiency while maintaining procedural sterility.

The training paradigm for R-PCI must also adapt. The learning curve associated with robotic systems has been shown to impact procedural duration and fluoroscopy use, especially in low-volume or inexperienced centers [17,23]. Future directions include the development of standardized robotic PCI training curricula, formal credentialing programs, and AI-powered procedural simulators that replicate tactile and visual feedback for complex cases. These initiatives could accelerate operator proficiency and promote consistent clinical outcomes. A particularly promising frontier lies in telestenting and remote robotic PCI, which can potentially expand access to expert coronary care in underserved and geographically remote regions. Pilot programs have already demonstrated the feasibility of remote PCI execution via high-speed networks, with the operator located outside the cath lab [39,46]. Future efforts must ensure secure communication protocols, real-time control precision, and on-site support for device loading and troubleshooting. The synergy between robotics, AI, and cloud-based procedural navigation may ultimately redefine the boundaries of interventional cardiology. Finally, health economics and sustainability will be critical to justify widespread institutional adoption. Future research should focus on cost-effectiveness models that incorporate reduced occupational injury, increased operator longevity, minimized PPE usage, and radiation-related savings. As the field moves toward zero-radiation procedural goals, robotic PCI must be positioned not only as a technological innovation but also as a value-based, ethically grounded standard of care.

## 3. Conclusions

Robotic-assisted PCI (R-PCI) represents a pivotal advancement in the evolution of interventional cardiology, combining mechanical precision with enhanced operator safety and the potential for significantly reduced radiation exposure. Over the past decade, substantial progress has been made in refining R-PCI platforms, integrating advanced imaging modalities, and implementing novel shielding strategies. These innovations not only mitigate occupational hazards but also lay the groundwork for safer, more sustainable procedural environments. Clinical studies consistently demonstrate that R-PCI achieves technical and clinical success rates comparable to manual PCI, while offering substantial reductions in radiation exposure to both patients and operators. Additionally, the integration of CT-derived planning, low-dose angiographic protocols, and advanced shielding systems supports a paradigm shift toward precision-guided, minimally irradiating interventions. Despite current limitations—including restricted device compatibility, lack of tactile feedback, and the need for manual intervention in complex cases—continued technological refinement and targeted operator training are bridging these gaps. Looking forward, the convergence of robotics with artificial intelligence, intravascular imaging, and remote capabilities may redefine procedural planning and execution. The trajectory of R-PCI suggests a movement not merely toward enhanced efficiency, but toward a reimagined standard of care—one that prioritizes operator health, patient safety, and procedural excellence. As evidence continues to accumulate and robotic platforms become more versatile, R-PCI should be embraced not only as a novel technology but as a cornerstone of value-based, ethically responsible interventional practice. Achieving radiation-free PCI may no longer be a theoretical aspiration, but an attainable goal—one grounded in innovation, data, and a growing commitment to protecting those who deliver cardiovascular care, hopefully in the not-so-distant future.

## Figures and Tables

**Table 1:** Overview of major clinical trials in robotic PCI.

Study (Year)	Design & Sample Size	Population/Lesion Type	Primary Endpoint	Radiation Reduction/Clinical Success	Key Limitations
**PRECISE (2013) [10]**	Prospective multicenter trial, *n* = 164	Single-lesion PCI	Technical & clinical success	95% ↓ operator dose	Limited to first-gen CorPath 200; no complex lesions
**CORA-PCI (2017) [11]**	Prospective registry, *n* = 315	Complex B2/C lesions	Clinical & technical success	93–100% clinical, 81–98.8% technical	Learning curve; lack of atherectomy and limited device compatibility
**Smitson et al. (2018) [14]**	Prospective single-center, *n* = 40	77.8% B2/C lesions	Technical success	90% technical, 97.5% clinical	Device delivery failures in calcified lesions
**Hirai et al. (2020) [15]**	Retrospective single-center, *n* = 51 CTOs	CTO PCI (hybrid robotic-manual)	Procedural completion	98% completed robotically	All CTOs crossed manually first
**R-EVOLUTION (2023) [16]**	Multicenter registry, *n* = 62	De novo lesions, radial access	Technical & clinical success	95.2% technical, 100% clinical	Limited sample size; early experience phase
**Patel et al. (2020) [22]**	Propensity-matched cohort, *n* = 560	Mixed lesion types	Radiation exposure	↓ air kerma & DAP in R-PCI (*p* < 0.01)	Observational; potential residual confounding

**Table 2:** Summary of radiation reduction trials and devices.

Study/Device	Type	Radiation Reduction	Key Features
**REDUCE-PCI [36]**	Clinical Trial	↓ 45% operator dose	Real-time dosimetry, staff education, and shielding optimization
**Gupta et al. 2021 [37]**	Clinical Trial	↓ 80% total dose	Low-frame-rate fluoroscopy (3.8 + 7.5 fps), reduced cine
**PROTECTION VIII [38]**	Multicenter Registry	↓ 47% median DAP	Flat-panel detectors, optimized collimation, default low-dose settings
**R-EVOLUTION Trial [16]**	Clinical Trial (Robotic PCI)	↓ 69% operator, ↓ 31% patient dose	Robotic PCI system (R-One platform), cockpit control, improved shielding
**Rampart IC [34]**	Shielding Device	↓ 99.7% staff exposure	Lead-acrylic mobile full-body enclosure, apron-free configuration
**Protego System [32]**	Shielding Device	↓ 95.9–99.8% (thyroid, waist)	Apron-free rigid and flexible barrier system for operator and assistant
**StemRad MD [26]**	Wearable Exoskeleton Shield	↓ >90% (brain, eyes, thyroid)	Bismuth-antimony core, orthopedic support, full-body wearable protection

**Table 3:** Contrast volume and procedural time.

Study	Contrast Use (R-PCI vs. M-PCI)	Procedural Time (R-PCI vs. M-PCI)	Statistically Significant?
**Smilowitz et al. [9]**	121 ± 47 mL vs. 137 ± 62 mL	44 ± 32.7 vs. 61 ± 19 min	No
**Madder et al. [12]**	167 ± 89 mL vs. 145 ± 92 mL	55 ± 22 min vs. 45 ± 37 min	Yes (time only)
**CORA-PCI [11]**	183.4 ± 78.7 vs. 202.5 ± 74 mL	43 ± 26 min vs. 34 ± 17 min	Yes (time)
**Hirai et al. [15]**	111 ± 39 mL vs. 118 ± 53 mL	89.6 ± 27.1 vs. 93.4 ± 30.5 min (NS)	No (contrast, time)
**Patel et al. [22]**	140 vs. 130 mL (NS)	37 vs. 27 min (*p* < 0.0005)	Yes (time only)
**R-EVOLUTION [16]**	—	↓ time with experience (>5 R-PCI cases)	Yes

**Table 4:** Clinical and technical success rates of robotic PCI platforms.

Study	System	Population	Technical Success (%)	Clinical Success (%)	Conversion to M-PCI (%)
**CORA-PCI [11]**	CorPath GRX	Mixed lesion complexity	97.6–98.8	100	5.5
**PRECISE [10]**	CorPath 200	Single-lesion PCI	91.7–98.8	97.6–100	~4
**Smitson et al. [14]**	CorPath GRX	77.8% B2/C lesions	90	97.5	10
**Hirai et al. [15]**	CorPath GRX	CTO (hybrid R-PCI)	98	100	2
**R-EVOLUTION [16]**	R-One (Robocath)	de novo stenosis, radial	95.2	100	3.2
**Patel et al. [22]**	CorPath GRX	Matched cohort (*n* = 560)	—	—	Radiation outcomes only

## Data Availability

The original data presented in the study are openly available in PubMed. Narrative review; no new data created.

## Abbreviations

The following abbreviations are used in this manuscript:

AI	Artificial intelligence
CCTA	Coronary computed tomography angiography
CTO	Chronic total occlusion
DAP	Dose area product
FFR	Fractional flow reserve
FFR-CT	CT-derived fractional flow reserve
fps	Frames per second
GRX	CorPath GRX robotic platform
IVUS	Intravascular ultrasound
iFR	Instantaneous wave-free ratio
M-PCI	Manual percutaneous coronary intervention
OCT	Optical coherence tomography
PCI	Percutaneous coronary intervention
R-PCI	Robotic percutaneous coronary intervention
STEMI	ST-elevation myocardial infarction
